# Interactions across Multiple Stimulus Dimensions in Primary Auditory Cortex

**DOI:** 10.1523/ENEURO.0124-16.2016

**Published:** 2016-09-01

**Authors:** David C. Sloas, Ran Zhuo, Hongbo Xue, Anna R. Chambers, Eric Kolaczyk, Daniel B. Polley, Kamal Sen

**Affiliations:** 1 Hearing Research Center and Department of Biomedical Engineering, Boston University, Boston, Massachusetts 02215; 2 Department of Mathematics and Statistics, Boston University, Boston, Massachusetts 02215; 3 Eaton-Peabody Laboratories, Massachusetts Eye and Ear Infirmary, Boston, Massachusetts 02114, and; 4 Department of Otolaryngology, Harvard Medical School, Boston, Massachusetts 02115

**Keywords:** auditory, evolutionary algorithm, generalized additive model, integration, sensory

## Abstract

Although sensory cortex is thought to be important for the perception of complex objects, its specific role in representing complex stimuli remains unknown. Complex objects are rich in information along multiple stimulus dimensions. The position of cortex in the sensory hierarchy suggests that cortical neurons may integrate across these dimensions to form a more gestalt representation of auditory objects. Yet, studies of cortical neurons typically explore single or few dimensions due to the difficulty of determining optimal stimuli in a high dimensional stimulus space. Evolutionary algorithms (EAs) provide a potentially powerful approach for exploring multidimensional stimulus spaces based on real-time spike feedback, but two important issues arise in their application. First, it is unclear whether it is necessary to characterize cortical responses to multidimensional stimuli or whether it suffices to characterize cortical responses to a single dimension at a time. Second, quantitative methods for analyzing complex multidimensional data from an EA are lacking. Here, we apply a statistical method for nonlinear regression, the generalized additive model (GAM), to address these issues. The GAM quantitatively describes the dependence between neural response and all stimulus dimensions. We find that auditory cortical neurons in mice are sensitive to interactions across dimensions. These interactions are diverse across the population, indicating significant integration across stimulus dimensions in auditory cortex. This result strongly motivates using multidimensional stimuli in auditory cortex. Together, the EA and the GAM provide a novel quantitative paradigm for investigating neural coding of complex multidimensional stimuli in auditory and other sensory cortices.

## Visual Abstract

**Figure:**
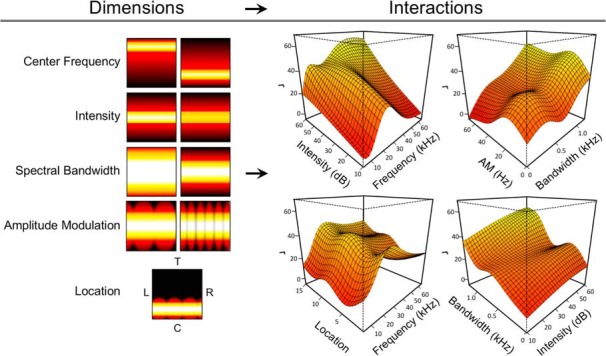

## Significance Statement

The auditory cortex is thought to be integral for the perception of complex sounds, which are characterized by multiple stimulus dimensions, such as center frequency, intensity, and bandwidth. Traditional studies of cortical neurons only consider one or few dimensions of sound at a time, but it is possible that cortical neurons integrate across these dimensions when processing sounds. Here, we apply an evolutionary algorithm and a generalized additive model to quantitatively explore cortical response to 5-dimensional auditory stimuli. Our results demonstrate that cortical neurons are significantly driven by interactions across stimulus dimensions in ways that are not captured by low-dimensional characterizations and motivate the use of multidimensional stimuli in the study of sensory cortices.

## Introduction

How does a sensory system recognize complex objects? This fundamental question in neuroscience remains poorly understood. Complex objects typically carry information about many stimulus dimensions. For example, an auditory object could be characterized by frequency, bandwidth, amplitude modulation, intensity, location, and other parameters. Given the importance of sensory cortex in the perception of objects and its place in the sensory hierarchy, it is likely to play an important role in integrating different stimulus dimensions represented separately in the periphery. For such neurons, the traditional approach of characterizing the neuron’s response to each dimension separately (e.g., a one-dimensional tuning curve) may be incomplete. Indeed, previous studies analyzing the separability of the spectrotemporal receptive field (STRF) of neurons have shown that cortical STRFs may not be separable (Depireux et al., 2001; Sen et al., 2001; Linden et al., 2003) and that cortical neurons can display nonlinear tone-tone interactions (Shamma et al., 1993; Nelken et al., 1994; Suga, 1994; Calford and Semple, 1995; Brosch and Schreiner, 1997; Sutter et al., 1999; Kadia and Wang, 2003). Thus, the use of multidimensional stimuli to probe cortex is likely to reveal important aspects of cortical processing and integration missed by traditional methods.

Evolutionary algorithms (EAs) have tremendous potential for investigating the neural coding of multidimensional stimuli in a wide variety of systems in neuroscience (Bleeck et al., 2003; Yamane et al., 2008; Carlson et al., 2011; Hung et al., 2012; DiMattina and Zhang, 2013; Chambers et al., 2014). EAs have been applied to investigate the coding of sounds in inferior colliculus and cochlear nucleus (Bleeck et al., 2003), 2-dimensional shapes in visual area V4 (Carlson et al., 2011), and 3-dimensional shapes in inferotemporal cortex (Yamane et al., 2008). Recently, an EA was developed for finding highly effective stimuli for maximizing the firing rate of auditory cortical neurons in a 5-dimensional stimulus space (Chambers et al., 2014). The EA probed cortical neurons with 5-dimensional stimuli and gradually modified these stimuli “online” based on the neural firing rate, to find stimuli that were most effective in driving the neuron. The parameter space in this situation is vast and impossible to explore using traditional methods. The EA was successfully able to identify highly effective stimuli for cortical neurons in five dimensions over several “generations” of stimuli.

Given the integrative role of auditory cortex, we hypothesized that cortical neurons show interactions across stimulus dimensions. Two important challenges arise in testing this hypothesis. First, traditional 1-dimensional tuning curves are insufficient, as interactions between dimensions must also be considered. Second, the use of multiple linear regression methods is not appropriate, given the nonlinear dependence of the cortical response on stimulus dimensions (e.g., nonlinear level tuning). Thus, an appropriate method should blend the ability to model interactions possessed by traditional linear models with nonlinear tuning curves. Here, we apply the generalized additive model (GAM) to address these issues. The GAM is a powerful method for performing nonlinear regression in statistics (Hastie and Tibshirani, 1991; Hastie et al., 2011). It can be thought of as an extension of the linear model (LM) and the generalized linear model (GLM) to allow a more flexible nonlinear dependence on stimulus dimensions. While the LM and the GLM have been widely applied to characterize sensory neurons (Klein et al., 2000; Theunissen et al., 2000; Depireux et al., 2001; Theunissen et al., 2001; Calabrese et al., 2011), the GAM remains underutilized in neuroscience. The objective of this study is to demonstrate the capacity of the GAM to uncover interactions between stimulus dimensions that cannot be revealed by more traditional 1-dimensional tuning curves, and to quantitatively characterize such interactions.

## Materials and Methods

### Experimental data

The quantitative methods in this paper were applied to experimental data collected by Chambers et al. (2014). An EA was used for online stimulus optimization based on single-unit spike feedback in the primary auditory cortex (A1) of awake, passively listening mice. Multichannel silicon probes (Neuronexus) were surgically implanted into A1 (located with functional mapping to reveal the characteristic tonotopic gradient) of male BCA/CaJ mice 8-10 weeks of age. At least 48 hours after implantation, the mice underwent recording sessions, during which a series of acoustic stimuli were presented by the EA and the responses from a well-isolated single unit from one of the 16 channels were recorded and analyzed online in order to drive the algorithm. The EA explored an acoustic space consisting of the following: center frequency (CF; 4-64 kHz in 0.1 octave increments), intensity (I; 10-60 dB in 10 dB increments), spectral bandwidth (BW; pure tone, 1.25 octave band in 0.25 octave increments), sinusoidal amplitude modulation frequency (AM; unmodulated [0 Hz], 70 Hz in 10 Hz increments), and speaker location (L; all permutations of left, right, top, and center). Acoustic stimuli were presented for 400 ms with 600 ms between each, and the firing rate was calculated during the entire stimulus window (0-400 ms). Each run of the EA search procedure began with 50 stimuli selected at random from the pool of 177,120 potential stimuli. Each stimulus was presented twice during the session. At the end of each generation of 50 stimuli, the stimuli were rank-ordered with respect to firing rate responses. The top 10 were used as “breeders” for the next generation such that their “offspring” were created by randomly shifting at least one acoustic dimension to a neighboring value. The most effective stimulus from the first generation was termed the “yardstick” and was repeated in all subsequent generations to estimate the effect of adaptation across generations. After the first generation, each generation consisted of 39 breeder-based, 1 yardstick, and 10 randomly selected stimuli from the stimulus pool to avoid focusing on local maxima. The maximum response magnitude was the maximum value of the firing rate over 6 generations of the EA for each neuron. For some single units, two runs of the EA were performed in order to compare the convergence from independent starting points. Results by Chambers et al. (2014) successfully showed the ability of EAs to converge on stimuli that maximized the firing rate. The methods in this paper further analyze the data gathered by the EA by quantifying the relationship between the stimulus dimensions and the neural response. Further experimental details can be found in Chambers et al. (2014).

### GAM

The GAM can be thought of as an extension of the LM and the GLM that allows a more flexible nonlinear dependence on stimulus dimensions. Here, the GAM is used to express average neural firing rate *r* as a sum of nonlinear functions. In this study, 15 possible nonlinear function terms are considered: 5 terms corresponding to individual stimulus dimensions (CF, I, BW, AM, and L) and their 10 possible combination pairs, defined as “interaction terms.” This may be represented as follows:

$$r=\beta_{0}+f_{1}(x_{1})+\cdots +f_{5}(x_{5})+f_{6}(x_{1},\;x_{2})+\cdots +f_{15}\left(x_{4},\;x_{5}\right)$$

where the predicted neural response *r*, assumed to have a normal distribution, is related to predictor variables *x_i_*and their unique pairs. Here, *x_i_* represents 1 of the 5 stimulus dimensions, and *f_i_* is a smooth function, with each of the 15 *f* terms individually determined.

### Model selection

A GAM was developed for each of *n* = 50 neurons using an iterative algorithm. GAM model training was performed on all stimuli presented during the EA. First, we performed an exhaustive search of all 32 possible models consisting only of individual stimulus dimensions. Because each of these models had a distinct number of free parameters, we used the Akaike Information Criterion (AIC) to determine the best model (Wood, 2006). In model selection, AIC considers the tradeoff between goodness of fit and complexity, with a lower AIC value indicating a better model. The lowest AIC value was used to determine the best of 32 GAMs containing 1-5 “main” dimensions. We then expanded this initial GAM by testing GAMs that contained these main dimensions and all of their possible interaction terms (up to 1024 possible models). From this family of possible models for each neuron, we selected the final overall best GAM as the one with the lowest AIC value. The median adjusted *r*^2^ value of the 50 final GAMs was 0.50, with values ranging from 0.11 to 0.89.

### Significant dimensions and interactions

For each neuron’s final GAM, the significance of the contribution of each term was analyzed. Significant terms were defined as those with *p* < 0.05. The number of significant interaction terms and the total number of significant terms were quantified for each neuron. The significant terms were summarized using a matrix where entries on the diagonal indicate the significance of main dimensions and entries on the upper triangle indicate the interaction terms. To quantify the contribution of interaction terms to the overall accuracy of a neuron’s GAM, the interaction terms were subtracted from the model, and the adjusted *r*^2^ value was recalculated.

### Visualization

A neuron’s entire “response space” was visualized by either a 1-dimensional “response curve” or a 2-dimensional “response surface.” One-dimensional response curves were generated by holding all other dimensions at their median values. Confidence intervals on 1-dimensional plots were defined as ±2× the SE. To visualize interactions, we plotted a neuron’s 2-dimensional response surfaces, fixing all other dimensions at their median values. The response surface effectively expresses the firing rate as a function of two main dimensions and one interaction term as follows:

$$r=\beta_{0}+f_{1}(x_{1})+f_{2}(x_{2})+f_{3}\left(x_{1},\;x_{2}\right)$$

GAM fitting and visual analysis were performed using the *mgcv* package in R (Wood, 2006). This software constrained the response spaces to be smooth using penalized regression splines and generalized cross-validation.

### Quantifying adaptation

Because the EA progressively converges on a single optimum stimulus, it is possible that the accuracy of the GAMs may be impacted by adaptation. In evaluating this possibility, we quantified adaptation as the percent difference in neural firing rate between the first and final presentations of the yardstick stimulus. Adaptation to the yardstick stimulus was a 46.4 ± 36.4% decrease in firing rate.

## Results

### Presence of significant interaction terms in A1

**Figure 1.**
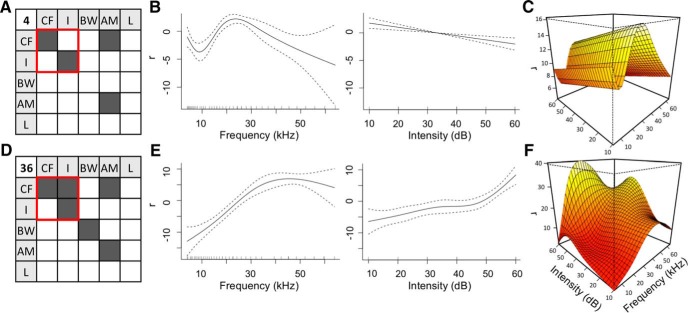
Presence of an interaction term. Significance matrices, 1-dimensional response curves, and CF-I response surface of (***A-C***) Neuron #4 and (***D-F***) Neuron #36. ***A***, ***D***, Significance matrices represent significant (*p* < 0.05, shaded dark) individual dimensions on the diagonal and interaction terms in the upper triangle. (The lower triangle is symmetric to the upper triangle and not shaded.) Red box represents the terms being considered in the 1- and 2-dimensional visualizations. ***B***, ***E***, One-dimensional response curve of each neuron with respect to CF (left) and I (right). Dashed lines indicate the confidence interval as ±2× SE. ***C***, ***F***, CF-I response surface of each neuron.

Figure 1 shows an example comparison between two neurons and their dependency on CF and I. Although both neurons are significantly driven by CF and I as individual dimensions, only the second (Fig. 1*D-F*) is impacted by a significant interaction term between these two. The first neuron appears to follow a tuning curve with respect to frequency and an inverse relationship with intensity (Fig. 1*B*), and these 1-dimensional trends are reflected in the 2-dimensional response surface (Fig. 1*C*). However, with the inclusion of an interaction term, the response surface of the second neuron does not have such obvious relationships to its individual dimensions (Fig. 1*E*,*F*).

**Figure 2.**
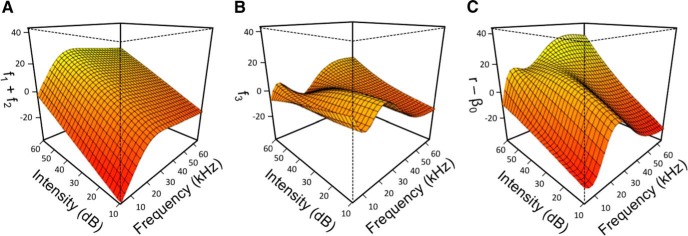
Example impact of interaction term on neuron response space. The CF-I response surface of an example neuron is visually separated into three components. For the expression, *r* = β_0_ + *f_1_(x_1_)* + *f_2_(x_2_)* + *f_3_(x_1_, x_2_)*, *x_1_* is CF, *x_2_* is I, *f_1_* and *f_2_* are individual dimension terms, and *f_3_* is an interaction term. ***A***, Individual dimension terms (*f_1_* + *f_2_*). ***B***, Interaction term (*f_3_*). ***C***, Neuron response offset by intercept (*r –* β_0_): that is (***A***) + (***B***) = (***C***).

**Figure 3.**
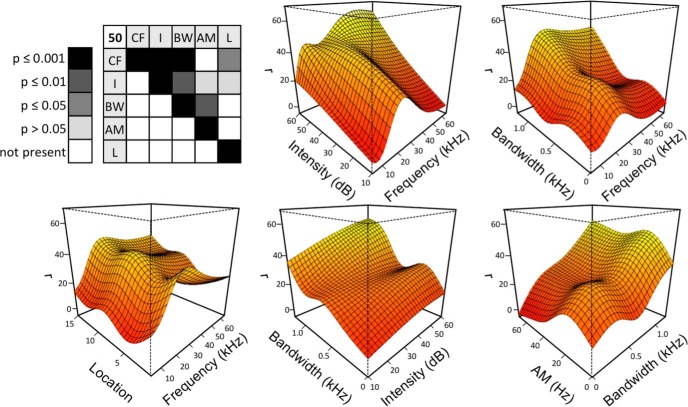
A cortical neuron with multiple significant interaction terms. The significance matrix (top left) of Neuron #50 represents 5 significant interaction terms (*p* < 0.05). The response surfaces of these 5 terms are shown as follows: CF-I, CF-BW, CF-L, I-BW, and BW-AM.

Figure 2 further explores the impact of an interaction term on a neuron’s response space. We separated the firing rate of a single neuron whose GAM has an interaction term between CF and I into individual dimension terms and an interaction term, which add together to create the overall modeled response. Inclusion of an interaction term appears to capture complexities of the neuron’s response that are missed when only individual dimensions are considered. To quantify this, adjusted *r*^2^ values are calculated for each GAM and then recalculated after dropping all interaction terms from this GAM. Such a removal of interaction terms causes a decrease in adjusted *r*^2^ from 0.50 ± 0.19 to 0.29 ± 0.16 with a median decrease in accuracy of 37.4% (data not shown). As shown in Figure 3, A1 neurons have the potential to exhibit dependence on many interaction terms, which highlights a level of complexity of neuronal response that cannot be captured when only considering individual dimensions.

### Population responses

**Figure 4.**
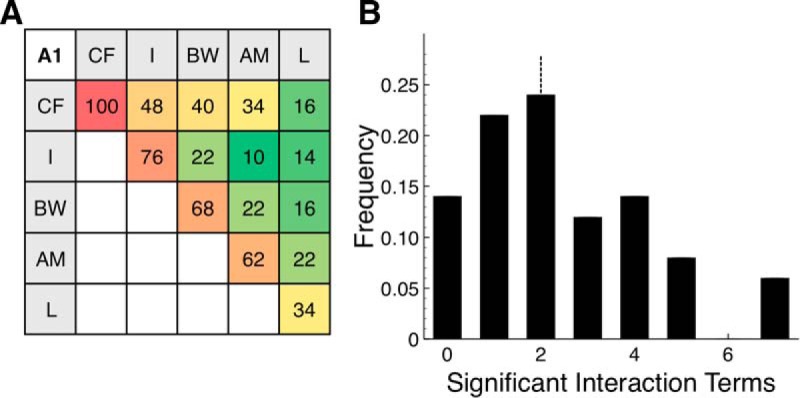
Population analysis of A1 neurons. ***A***, Percent of neurons driven by each significant term. Red represents highest value (100%). Green represents lowest value (10%). Lower triangle is symmetric to upper triangle and is left blank. ***B***, Histogram of number of significant interaction terms per neuron. Median number of significant interaction terms was 2, shown by dashed vertical line.

Figure 4 summarizes the dependence of A1 responses across the population. Of the 5 individual dimension terms considered, CF was the most common, significantly modulating the firing rate of 100% of sample neurons. This was followed by I (76.0%), BW (68.0% each), AM (62.0%), and L (34.0%) (Fig. 4*A*). GAMs produced for A1 neurons had 2.4 ± 1.9 interaction terms and 5.8 ± 2.6 total significant terms (Fig. 4*B*). Of the 50 neurons analyzed, only 7 had no significant interaction terms.

## Discussion

EAs have long been recognized as a potentially powerful tool and applied successfully to characterize sensory processing of multidimensional stimuli in a handful of studies (Bleeck et al., 2003; Yamane et al., 2008; Carlson et al., 2011; Hung et al., 2012; DiMattina and Zhang, 2013; Chambers et al., 2014). However, some significant barriers have prevented the widespread use of EAs. First, it is not entirely clear whether it is strictly necessary to probe the full multidimensional space of stimuli (e.g., using an EA) or whether adequate information can be obtained by exploring each dimension individually. Second, quantitative methods for analyzing the multidimensional data sets resulting from the EA are lacking. In this study, we applied the GAM to address these issues.

Previous quantitative methods for characterizing cortical responses have included the LM and GLM. A specific instance of the LM, the STRF, has been applied extensively to investigate cortical responses (Klein et al., 2000; Theunissen et al., 2000, 2001; Depireux et al., 2001; Calabrese et al., 2011), and the GLM has extended this approach (Calabrese et al., 2011). Other studies have indicated that cortical STRFs may not be separable (Depireux et al., 2001; Sen et al., 2001; Linden et al., 2003) and that cortical neurons can display nonlinear tone-tone interactions (Shamma et al., 1993; Nelken et al., 1994; Suga, 1994; Calford and Semple, 1995; Brosch and Schreiner, 1997; Sutter et al., 1999; Kadia and Wang, 2003), suggestive of interactions across multiple stimulus dimensions. Although these studies have revealed aspects of cortical processing, they have been limited in two important ways. First, these studies did not quantify interactions across more than 2 stimulus dimensions explicitly. Second, the LM and GLM are limited in their ability to capture strong and diverse patterns of nonlinearities evident in cortical neurons (Theunissen et al., 2000; Bar-Yosef et al., 2002; Machens et al., 2004; Sadagopan and Wang, 2009). These limitations can be surpassed, in principle, by using information theoretic techniques for characterizing cortical neurons, such as maximally informative dimensions (Sharpee et al., 2004; Atencio et al., 2008, 2009,2012). However, this approach has the disadvantage that it is highly data intensive and limited by the “curse of dimensionality” (i.e., the difficulty of searching for optimal stimuli in a high dimensional stimulus space) and is therefore difficult to apply for multidimensional stimuli. Thus, there continues to be a need in the field for quantitative methods that are able to characterize the neural coding of multidimensional stimuli, including nonlinearities.

The GAM extends the LM and GLM by allowing the neural response (in our case, the average firing rate) of the neuron to be expressed as sum of nonlinear functions (Hastie and Tibshirani, 1991; Hastie et al., 2011). These nonlinear functions are constrained to be smooth but can otherwise be highly flexible in form. The importance of this feature of the GAM can be appreciated by considering the dependence of the response on the stimulus dimensions. For example, the dependence of the firing rate on frequency is highly nonlinear and can take a on a wide diversity of shapes in cortex. The dependence of firing rate on intensity can be quasilinear up to a certain level in some cases but is often sigmoidal or nonmonotonic over the relevant range of intensities. The GAM allows a flexible way to model such relationships. Moreover, the GAM also allows modeling interactions between stimulus dimensions, by including interaction terms (see Materials and Methods).

The GAM results confirmed familiar aspects of cortical responses but also revealed novel aspects. For example, we found that all neurons in our dataset were significantly modulated by frequency, consistent with the fact that frequency is a fundamental dimension for cortical responses (Merzenich et al., 1975; Guo et al., 2012). We also found that, while many cortical neurons were sensitive to sound intensity, a proportion of cortical neurons in our data were not significantly modulated by intensity, consistent with results showing the emergence of neurons robust to intensity variations at the cortical level (Billimoria et al., 2008; Sadagopan and Wang, 2008; Chambers et al., 2014). In addition to these 2 fundamental dimensions, cortical neurons also showed sensitivity to BW, AM, and L, with decreasing proportions of cortical neurons sensitive to each.

Most importantly, the GAM revealed that cortical neurons can be sensitive to interactions across stimulus dimensions. On average, cortical neurons were sensitive to ∼3 interactions across different stimulus dimensions in a 5-dimensional stimulus space. These results indicate that low dimensional characterizations of cortical neurons, which explore 1 or 2 dimensions, while fixing others to arbitrary values, are likely to miss important aspects of cortical response. This strongly motivates the use of EA in characterizing responses in sensory cortex using multidimensional stimuli.

A potential challenge facing EAs is adaptation in firing rate. Thus, adaptation may also have influenced the quality of the GAM. However, we found no significant correlation between the amount of adaptation and the performance (adjusted *r*^2^) of the GAM (*p* = 0.152, correlation coefficient = 0.2059). One potential explanation is that, with the exception of a single “yardstick” stimulus, stimuli in the EA were rarely repeated exactly. Previous studies have shown that adaptation in auditory cortex can be highly stimulus specific (Nelken, 2014). This may have mitigated the effects of adaptation on the EA and GAM.

Overall, our results suggest that primary auditory cortex integrates information across multiple stimulus dimensions both at the single neuron level, through multiple interactions within single neurons, and at the population level, through a diverse range of interactions across different neurons. Object formation likely requires several hierarchical steps to accomplish. Although this process likely is not completed within A1, the interactions we observed may be a key computation toward object formation. Future experimental and theoretical studies investigating the synaptic and network mechanisms underlying interactions, and the impact of interactions on single neuron and population coding should clarify whether and how such interactions contribute to the cortical substrate for complex object recognition.

### Future Directions

Several additional future directions merit further exploration. First, the GAM analysis performed here was run on the collected dataset using the EA after the experiments were complete. In the future, it would be interesting to apply the GAM online as the data are collected. Second, the GAM and EA were applied to a specific family of 5-dimensional stimuli. In principle, this approach could be applied to any family of sounds that can be parameterized systematically. For example, a similar paradigm could be used with parametrically specified multitone combinations or ripple stimuli. However, it remains unclear how high the dimensionality of the stimulus space can be to still remain tractable for exploration with the EA, within typical time limits for data collection. Third, the GAM as applied here takes into account encoding by the firing rate only ignoring temporal structure in the responses. The vast majority of literature on GAMs is for static inputs, with a few extensions for dynamic inputs. In the future, it would be interesting to extend the GAM to account for temporal structure in neural responses.

## Synthesis

The decision was a result of the Reviewing Editor Tatyana Sharpee and the peer reviewers coming together and discussing their recommendations until a consensus was reached. A fact-based synthesis statement explaining their decision and outlining what is needed to prepare a revision is listed below. The following reviewer(s) agreed to reveal their identity: Stephen David

The reviewers agreed that the manuscript advanced the field by providing new approaches for characterizing the selectivity of auditory neurons. Reviewers listed specific suggestions for improving the manuscript, including adding discussions of STRF separability, the role of spike timing, and further details of of the fitting process. Please revise the manuscript accordingly.

Reviewer 1:

This study demonstrates a new approach to modeling filter properties of neurons in the auditory system, using a generalized additive model. The GAM describes neural responses as the sum of first- and second-order non-parametric functions of multiple stimulus dimensions (frequency, level, bandwidth, etc.) and can be fit using evolutionary algorithms. The authors demonstrate its feasibility on a data set collected from mouse A1. Especially critical to this demonstration is a model selection process by which they constrain the number of model free parameters, which is necessary to make the fit tractable.

The new approach is interesting and worth publishing as a new method. However, several critical features of the study are not described in enough detail.

1. (p. 2) It is true that one-dimensional tuning curve analysis is common and the authors' point that its descriptive power is limited is quite reasonable. However, the current study seems to ignore a fairly substantial literature that has made this point at several levels. First, there has been investigation into the separability of linear STRFs, and it is generally agreed that STRFs are not separable (Simon .. &amp; Shamma 2007). Second, several studies have identified nonlinear tone-tone interactions, which of course are out of the scope of the current model, but seem relevant (eg, Brosch &amp; Schreiner 1997). These points are touched on a bit in the discussion but some mention seems important for the introduction.

2. (p. 3) Were the data collected using an EA for fitting the GAM? Or for fitting the lower dimensional model cited in Chambers et al 2014? The way the EA is described, it appears that it should be run iteratively during data collection, but the GAM analysis appears to have been run after experiments were complete. The ambiguity makes the reader wonder whether the fits are necessarily optimal and/or if running the EA online is an important part of fitting the GAM. A clarification on how the data were collected and if the on-line EA is really important for the current results would clear this up.

3. (p. 4) "dependence of firing rate on frequency" This is cited as a nonlinearity, which may be technically correct for some class of models. But it seems very confusing when most tuning curve models can in fact account for arbitrary frequency tuning. Would nonlinear level tuning be a better example to cite?

4. (p. 5) What window of time relative to each stimulus was used to calculate firing rate? The whole stimulus period? Sustained period?

5. (pp. 6, 8) It is not clear from the text how response curves were constrained to be smooth. Was a hyperparameter required? Or was this obtained by the cited fit algorithm?

6. (p. 9) Was the model with no interaction terms (r=0.29 average performance) optimized for that model domain or did it simply have the interaction terms removed? I.e., was this equivalent to the model fit in Chambers et al?

7. (p. 10) Again some mention of separability seems appropriate re: STRFs.

8. (p. 12) A possible limitation of the GAM is that it appears to only account for one sparse stimulus at a time in the 5-D stimulus space. For example, can it model responses to a chord constructed from multiple tones at different frequencies or to a more complex natural sound? This should be discussed relative to other modeling approaches, such as the GLM.

Reviewer 2:

This report extends a previous analysis of auditory cortical responses using an Evolutionary Algorithm. The algorithm is able to systematically explore multiple acoustic features, and determine the combination that best drives a neuron in terms of firing rate. The authors apply an established statistical learning technique, the General Additive Model (GAM), to determine if the features that were found interact with each other. This is interesting, since if the features do not interact then each one may be found relatively independently of the others. If there are dependencies, then combinations of features need to be explored, and characterizing a neuron becomes more difficult. The authors show that many neurons are sensitive to feature interactions.

In the following, I mainly list some aspects that the authors may wish to consider and elaborate upon. The methods that were applied appear sound and appropriately implemented.

- Abstract/Introduction: This is a matter of taste, but stating that sensory cortex is responsible for perception may be interpreted in many ways. The idea of objects tends to evoke thoughts of generalization beyond acoustic features, but this does not seem to be what occurs in AI, since the EA algorithm converges on one set of features. Some more specificity regarding this concept may be appropriate.

- Introduction: "optimal stimulus" is also a loaded term. Optimal needs to be defined in terms of what is being optimized. In this case, optimal merely means that the stimulus evokes the largest firing rate, though spike timing, which may be important, is not accounted for.

- How much explanatory power is lost when only one-dimensional tuning curves are used compared to the full model?

- How much explanatory power is lost when interactions are included/not included? The authors show that many interactions are significant. Can they provide a measure for the amount of variance accounted for that is lost if interactions are ignored?

- Consider changing "Chambers et al." to "Chambers et al. (2014)".

- Page 5: from where is the maximum response magnitude obtained? Is it from the "yardstick" stimulus?

-Page 6: change "average neural firing r rate" to "the average neural firing rate, r," or something similar

- Page 6: how many observations were in the GAM model training? Is it all presented stimuli?

- The analysis assumes a rate code. Do the authors have thoughts for the discussion on the amount of information that might be due to spike timing, or how their approach could take this into account?

- Page 8: regarding adaptation, as the EA converges, do stimuli become more similar? The possibility of SSA is noted later on pages 12-13, so it would be helpful to note if the firing rate merely stabilized/plateaued, or decreased as the EA converged. This would also give information on any habituation that took place.

-Do the authors have thoughts on whether an STRF that is found by presenting noise-like stimuli may converge faster to the optimal parameters than the EA?

- It would be helpful for readers to have axes on all figures more clearly notated with tickmarks and numbers (ex: Page 20, C,F). Some interpretive labeling/description for the y-axes on page 20.

- Page 23: What may be the cause for the small number of Location and Intensity interaction terms?

## References

[B1] Atencio CA, Sharpee TO, Schreiner CE (2008) Cooperative nonlinearities in auditory cortical neurons. Neuron 58:956-966. 10.1016/j.neuron.2008.04.026 18579084 PMC2535914

[B2] Atencio CA, Sharpee TO, Schreiner CE (2009) Hierarchical computation in the canonical auditory cortical circuit. Proc Natl Acad Sci U S A 106:21894-21899. 10.1073/pnas.0908383106 19918079 PMC2799842

[B3] Atencio CA, Sharpee TO, Schreiner CE (2012) Receptive field dimensionality increases from the auditory midbrain to cortex. J Neurophysiol 107:2594-2603. 10.1152/jn.01025.2011 22323634 PMC3362274

[B4] Bar-Yosef O, Rotman Y, Nelken I (2002) Responses of neurons in cat primary auditory cortex to bird chirps: effects of temporal and spectral context. J Neurosci 22:8619-8632. 12351736 10.1523/JNEUROSCI.22-19-08619.2002 PMC6757805

[B5] Billimoria CP, Kraus BJ, Narayan R, Maddox RK, Sen K (2008) Invariance and sensitivity to intensity in neural discrimination of natural sounds. J Neurosci 28:6304-6308. 10.1523/JNEUROSCI.0961-08.2008 18562600 PMC2730838

[B6] Bleeck S, Patterson RD, Winter IM (2003) Using genetic algorithms to find the most effective stimulus for sensory neurons. J Neurosci Methods 125:73-82. 10.1016/S0165-0270(03)00040-2 12763233

[B7] Brosch M, Schreiner CE (1997) Time course of forward masking tuning curves in cat primary auditory cortex. J Neurophysiol 77:923-943. 9065859 10.1152/jn.1997.77.2.923

[B8] Calabrese A, Schumacher JW, Schneider DM, Paninski L, Woolley SM (2011) A generalized linear model for estimating spectrotemporal receptive fields from responses to natural sounds. PLoS One 6:e16104. 10.1371/journal.pone.0016104 21264310 PMC3019175

[B9] Calford MB, Semple MN (1995) Monaural inhibition in cat auditory cortex. J Neurophysiol 73:1876-1891. 7623087 10.1152/jn.1995.73.5.1876

[B10] Carlson ET, Rasquinha RJ, Zhang K, Connor CE (2011) A sparse object coding scheme in area V4. Curr Biol 21:288-293. 10.1016/j.cub.2011.01.013 21315595 PMC3070463

[B11] Chambers AR, Hancock KE, Sen K, Polley DB (2014) Online stimulus optimization rapidly reveals multidimensional selectivity in auditory cortical neurons. J Neurosci 34:8963-8975. 10.1523/JNEUROSCI.0260-14.2014 24990917 PMC4078078

[B12] Depireux DA, Simon JZ, Klein DJ, Shamma SA (2001) Spectro-temporal response field characterization with dynamic ripples in ferret primary auditory cortex. J Neurophysiol 85:1220-1234. 11247991 10.1152/jn.2001.85.3.1220

[B13] DiMattina C, Zhang K (2013) Adaptive stimulus optimization for sensory systems neuroscience. Front Neural Circuits 7:101. 10.3389/fncir.2013.00101 23761737 PMC3674314

[B14] Guo W, Chambers AR, Darrow KN, Hancock KE, Shinn-Cunningham BG, Polley DB (2012) Robustness of cortical topography across fields, laminae, anesthetic states, and neurophysiological signal types. J Neurosci 32:9159-9172. 10.1523/JNEUROSCI.0065-12.2012 22764225 PMC3402176

[B15] Hastie T, Tibshirani R (1991) Generalized additive models. London: Chapman & Hall. 10.1177/096228029500400302 8548102

[B16] Hastie T, Tibshirani R, Friedman J (2011) The elements of statistical learning, Ed 2. New York: Springer.

[B17] Hung CC, Carlson ET, Connor CE (2012) Medial axis shape coding in macaque inferotemporal cortex. Neuron 74:1099-1113. 10.1016/j.neuron.2012.04.029 22726839 PMC3398814

[B18] Kadia SC, Wang X (2003) Spectral integration in A1 of awake primates: neurons with single- and multipeaked tuning characteristics. J Neurophysiol 89:1603-1622. 10.1152/jn.00271.2001 12626629

[B19] Klein DJ, Depireux DA, Simon JZ, Shamma SA (2000) Robust spectrotemporal reverse correlation for the auditory system: optimizing stimulus design. J Comput Neurosci 9:85-111. 10946994 10.1023/a:1008990412183

[B20] Linden JF, Liu RC, Sahani M, Schreiner CE, Merzenich MM (2003) Spectrotemporal structure of receptive fields in areas AI and AAF of mouse auditory cortex. J Neurophysiol 90:2660-2675. 10.1152/jn.00751.2002 12815016

[B21] Machens CK, Wehr MS, Zador AM (2004) Linearity of cortical receptive fields measured with natural sounds. J Neurosci 24:1089-1100. 10.1523/JNEUROSCI.4445-03.2004 14762127 PMC6793584

[B22] Merzenich MM, Knight PL, Roth GL (1975) Representation of cochlea within primary auditory cortex in the cat. J Neurophysiol 38:231-249. 1092814 10.1152/jn.1975.38.2.231

[B23] Nelken I (2014) Stimulus-specific adaptation and deviance detection in the auditory system: experiments and models. Biol Cybern 108:655-663. 10.1007/s00422-014-0585-7 24477619

[B24] Nelken I, Prut Y, Vaadia E, Abeles M (1994) Population responses to multifrequency sounds in the cat auditory cortex: one- and two-parameter families of sounds. Hear Res 72:206-222. 8150737 10.1016/0378-5955(94)90220-8

[B25] Sadagopan S, Wang X (2008) Level invariant representation of sounds by populations of neurons in primary auditory cortex. J Neurosci 28:3415-3426. 10.1523/JNEUROSCI.2743-07.2008 18367608 PMC6670591

[B26] Sadagopan S, Wang X (2009) Nonlinear spectrotemporal interactions underlying selectivity for complex sounds in auditory cortex. J Neurosci 29:11192-11202. 10.1523/JNEUROSCI.1286-09.2009 19741126 PMC2757444

[B27] Sen K, Theunissen FE, Doupe AJ (2001) Feature analysis of natural sounds in the songbird auditory forebrain. J Neurophysiol 86:1445-1458. 11535690 10.1152/jn.2001.86.3.1445

[B28] Shamma SA, Fleshman JW, Wiser PR, Versnel H (1993) Organization of response areas in ferret primary auditory cortex. J Neurophysiol 69:367-383. 8459273 10.1152/jn.1993.69.2.367

[B29] Sharpee T, Rust NC, Bialek W (2004) Analyzing neural responses to natural signals: maximally informative dimensions. Neural Comput 16:223-250. 10.1162/089976604322742010 15006095

[B30] Suga N (1994) Processing of auditory information carried by species-specific complex sounds In: The cognitive neurosciences (Gazzaniga MS, ed), pp 295-313. Cambridge, MA: Massachusetts Institute of Technology.

[B31] Sutter ML, Schreiner CE, McLean M, O’Connor KN, Loftus WC (1999) Organization of inhibitory frequency receptive fields in cat primary auditory cortex. J Neurophysiol 82:2358-2371. 10561411 10.1152/jn.1999.82.5.2358

[B32] Theunissen FE, Sen K, Doupe AJ (2000) Spectral-temporal receptive fields of nonlinear auditory neurons obtained using natural sounds. J Neurosci 20:2315-2331. 10704507 10.1523/JNEUROSCI.20-06-02315.2000 PMC6772498

[B33] Theunissen FE, David SV, Singh NC, Hsu A, Vinje WE, Gallant JL (2001) Estimating spatio-temporal receptive fields of auditory and visual neurons from their responses to natural stimuli. Network 12:289-316. 11563531

[B34] Wood S (2006) Generalized additive models: an introduction with R. London: Chapman and Hall/CRC.

[B35] Yamane Y, Carlson ET, Bowman KC, Wang Z, Connor CE (2008) A neural code for three-dimensional object shape in macaque inferotemporal cortex. Nat Neurosci 11:1352-1360. 10.1038/nn.2202 18836443 PMC2725445

